# Association between change in self-efficacy and reduction in disability among patients with chronic pain

**DOI:** 10.1371/journal.pone.0215404

**Published:** 2019-04-16

**Authors:** Yusuke Karasawa, Keiko Yamada, Masako Iseki, Masahiro Yamaguchi, Yasuko Murakami, Takao Tamagawa, Fuminobu Kadowaki, Saeko Hamaoka, Tomoko Ishii, Aiko Kawai, Hitoshi Shinohara, Keisuke Yamaguchi, Eiichi Inada

**Affiliations:** 1 Department of Pain Medicine, Juntendo University Graduate School of Medicine, Bunkyo-ku, Tokyo, Japan; 2 Medical Affairs, Pfizer Japan, Shibuya-ku, Tokyo, Japan; 3 Department of Anesthesiology and Pain Medicine, Juntendo University Faculty of Medicine, Bunkyo-ku, Tokyo, Japan; 4 Department of Psychology, McGill University, Montreal, Quebec, Canada; 5 Department of Anesthesiology, The Jikei University School of Medicine, Minato-ku, Tokyo, Japan; Tokyo Metropolitan Institute of Medical Science, JAPAN

## Abstract

**Purpose:**

This study aimed to investigate whether changes in psychosocial factors and pain severity were associated with reduction in disability due to pain among patients with chronic pain. We hypothesized that increased self-efficacy would reduce disability.

**Patients and methods:**

This longitudinal observational study included 72 patients. Patients’ psychological and physical variables were assessed before and after 3 months of treatment. Demographic and clinical information were collected, including the Pain Disability Assessment Scale (PDAS), the Pain Self-Efficacy Questionnaire (PSEQ), the Hospital Depression and Anxiety Scale, and the Numeric Rating Scale (NRS) to assess pain intensity. First, univariate regression analyses were conducted to clarify associations between change in PDAS and sex, age, pain duration, changes in psychosocial factors (self-efficacy, anxiety, and depression) and change in pain intensity. Second, multivariate regression was conducted using the variables identified in the univariate analyses (PSEQ and NRS) to detect the most relevant factor for reducing disability.

**Results:**

Univariate regression analyses clarified that changes in PSEQ (β = −0.31; 95% CI: −0.54–−0.08, p = 0.008) and NRS (β = 0.24; 95% confidence interval [CI]: 0.01–0.47, p = 0.04) were associated with reduction in PDAS. Multivariate regression analysis demonstrated that change in PSEQ (β = 0.26; 95% CI: −0.50–−0.02; p = 0.01) was associated with a reduction in disability, independent of change in NRS.

**Conclusion:**

These findings suggest improved self-efficacy is associated with reduced disability in patients with chronic pain, independent of reduction in pain intensity. Focusing on improvement in self-efficacy may be an effective strategy in chronic pain treatment in addition to pain relief.

## Introduction

Chronic pain carries a serious social burden, and is an important global health issue [1] [2] [3]. For many individuals, chronic pain interferes with social life, especially because it negatively impacts activities of daily living (ADL) and health-related quality of life (QOL) [4] [5]. Moreover, chronic pain bears a high economic cost [6].

Chronic pain accompanied by negative psychosocial factors often hinders healthcare providers in achieving treatment goals. For example, the fear-avoidance model explains that negative psychosocial factors (e.g., catastrophizing, fear, and depression) have a possible role in mediating disability in patients with chronic pain [7]. Although medications such as nonsteroidal anti-inflammatory drugs (NSAIDs) and opioids are often used to eliminate pain, it is difficult to improve the psychosocial factors associated with chronic pain by medication only. In addition, NSAIDs often cause adverse gastrointestinal and cardiovascular events [8] [9]. Opioids also cause various adverse events, and over-use of opioids for non-cancer pain may result in narcotics addiction and induce an opioid crisis [10]. Completely eliminating pain is not a feasible strategy in chronic pain treatment; for example, Thomas and Lee noted that “zero pain is not the goal” [11]. Improvement in ADL should be a target in chronic pain treatment. Improving both ADL and QOL using a multimodal approach that includes rehabilitation, exercise therapy, cognitive behavioral therapy, and mindfulness is recommended in several chronic pain treatment guidelines [12] [13].

Several physical and psychological factors are thought to contribute to enhancing disability in patients with chronic pain. Pain intensity is related to physical activity [14] and is also reported to have a negative association with return to work in patients with chronic low back pain [15]. Depression is a common comorbid psychological condition in those with chronic pain, and is known to be a significant determinant of pain-related disability [14]. Anxiety often leads patients with chronic pain to avoid activities that can exacerbate pain and worsen disability [16]. Pain-related self-efficacy has been found to affect pain severity, negative psychological factors, and disability related to low back and shoulder pain [17] [18] [19]. Self-efficacy is also reported to mediate the relationship between pain intensity and disability [20] [21]. Moreover, increased self-efficacy was a significant predictor of positive change in health status through self-management programs among patients with arthritis [22]. To our knowledge, few cross-sectional studies have investigated the direct association between self-efficacy and disability in patients with chronic pain [23], and no longitudinal studies have been conducted in Asia, including Japan. A meta-analysis integrating the results of 83 studies that were conducted outside Japan showed that self-efficacy was associated with pain-related outcomes such as impairment, affective distress, and pain severity in various types of painful diseases [24]. The meta-analysis reported that several studies had longitudinally investigated the relationship between self-efficacy and disability among patients with specific site pain, such as low back or arthritis pain [17] [18] [19]. However, no studies targeted chronic pain, including neuropathic pain, headache, orofacial pain, and visceral pain. However, there were no studies that targeted chronic pain, including neuropathic pain, headache, orofacial pain, and visceral pain. For research purposes, chronic pain is usually categorized by pain site or cause of pain. However, widespread pain and duplicate causes of pain are likely to be misclassified in such categorization. Regardless of pain site or cause of pain, subjective persistent pain itself has a pathological meaning. Therefore, evaluation of all-inclusive chronic pain is required for comprehensive research. The present study aimed to examine the association between change in psychosocial factors and pain severity with reduction in disability among patients with chronic pain in a Japanese clinical pain care setting using a longitudinal approach.

## Methods

### Participants

**Fig 1:**
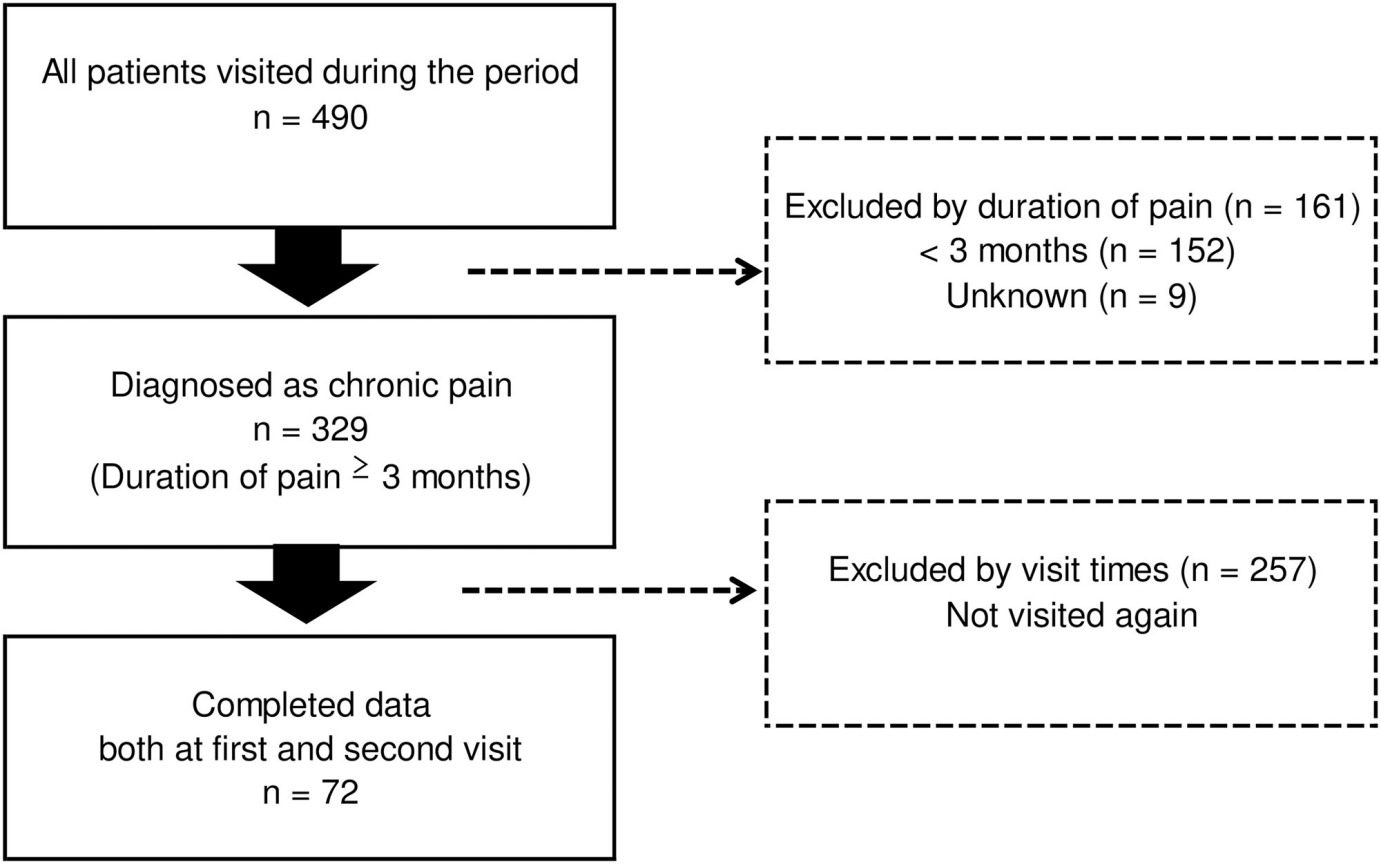
Diagram of the participant selection process.

We identified 490 outpatients from March 2016 to December 2016 in a pain clinic (Fig 1).

Individuals were eligible for participation if they: 1) were aged 20 years or older; 2) were able to read, write, and understand questionnaires written in Japanese; and 3) had persistent pain for at least 3 months. Participants completed four measures: the Pain Disability Assessment Scale (PDAS) [25], the Pain Self-Efficacy Questionnaire (PSEQ) [26], the Hospital Depression and Anxiety Scale (HADS) [27], and a Numeric Rating Scale (NRS) [28]. These measures were completed at pre- and post-treatment (3 months after the initial visit) evaluations. We did not control therapeutic interventions in the present study. In addition, we did not use a standardized treatment protocol, and physicians selected the most suitable therapy for each patient. Physicians conducted flexible treatment, which was based on standard clinical guidelines for chronic pain in Japan [13]. Treatment options included pharmacotherapy, interventional management, psychological approach, and rehabilitation [13]. The questionnaires were completed in the pain clinic waiting room. Demographic data, medical history, duration of pain, and diagnosis were collected from participants’ medical records. Physicians specialized in pain management categorized each participant using pain classifications of the International Classification of Diseases, 11th revision [29]. Of 490 identified patients, 161 with pain duration less than 3 months or unknown duration were excluded. A further 257 patients who did not visit the clinic again were also excluded. This left 72 participants (33 men and 39 women) with complete data on all variables for inclusion in the study. In this study, we did not identify reasons why more than half of the patients had not visited our clinic again at 3 months after their first visit. We assume that there may be various reasons for this, including: 1) patients improved and did not need any further medical care at 3 months; 2) patients improved and their physician referred them to a primary care doctor during the 3-month study period; and 3) patients discontinued therapy without notice.

### Ethical considerations

This study was approved by the Institutional Review Board for Clinical Research of Juntendo University Hospital (IRB No.17-234). Informed consent was obtained from all participants before they completed the measures.

### Measures

#### Pain-related disability

The PDAS is a 20-item tool that assesses the degree of impact of pain-related disability on a person’s lifestyle during the past week [25]. Respondents are asked to rate each activity on a Likert scale from 0 (“Pain did not interfere with this activity”) to 3 (“Pain completely interfered with this activity”) [25]. Total scores range from 0 to 60, with higher scores indicating greater levels of pain interference [25].

#### Pain-related self-efficacy

The PSEQ is a measure of generalized pain-related self-efficacy and assesses the degree of an individual’s confidence in performing a number of activities despite pain [26]. Each item is rated on a 7-point Likert scale from 0 (“not at all confident”) to 6 (“completely confident”). Total scores can range from 0 to 60, with higher scores indicating greater self-efficacy for performing despite pain. A previous study reported that a score over 40 points was associated with work restoration and functional improvement in patients with chronic upper limb pain, although a clear cut off value representing recovery from chronic pain has not yet been defined [30].

#### Anxiety and depression

The HADS is a self-report measure that was originally developed to assess anxiety and depression in clinical populations [27]. It comprises 14 items on two 7-item subscales (depression and anxiety), with each item rated on a scale from 0 to 3 according to how respondents’ felt recently. Respondents are scored from 0 and 21 for each subscale. A systematic review that included a large number of studies identified a cut-off point of 8 (of 21) as indicating possible anxiety or depression [31].

#### Pain intensity

The NRS is a common scale for quantifying pain, and is anchored by 0 (“no pain”) and 10 (“worst pain”) [28]. The scale is used for recording average pain intensity. The scores participants reported for pain experienced in the past 24 hours were used in this study [28].

### Statistical analysis

Statistical analyses were performed using EZR version 1.27 [32]. Participants’ demographic data were reported as means and standard deviations (SD), with the proportion of each classification as a percentage. Changes in each score at the first and second visits were analyzed using paired t-tests.

Univariate regression analyses were conducted to investigate which variables showed single associations with disability. Change in PDAS was considered an objective variable, with changes in PSEQ, HADS-Anxiety, HADS-Depression, and NRS considered as explanatory variables. Age, sex, and pain duration were variables used for adjustment. Subsequently, multivariate regression analysis was conducted to detect the most relevant factor for improving physical disability, using the identified explanatory variables. All statistical inferences were based on a significance level of P<0.05 (two-tailed tests).

## Results

### Participants’ characteristics

Demographic variables are shown in Table 1.

**Table 1:** Participants’ characteristics.

**Variables**	
Number of patients (Men/Women)	72 (33/39)
Age, years [mean (SD)]	65.2 (14.8)
Duration of disease, months [mean (SD)]	52.6 (86.4)
**Classification of chronic pain for ICD-11 candidate**	**Number (%)**
1.Primary Pain	7 (9.7)
2.Cancer Pain	2 (2.8)
3.Postsurgical and Posttraumatic Pain	9 (12.5)
4.Neuropathic Pain	45 (62.5)
5.Headache and Orofacial Pain	1 (1.4)
6.Viscaral Pain	1 (1.4)
7.Muscloskeletal Pain	7 (9.7)

SD, standard deviation.

### Changes in measures

All measures were significantly improved at the post-treatment evaluation (3 months after the initial visit) (Table 2).

**Table 2:** Changes in pain-related assessments at pre- and post-treatment.

	Pre treatment	Post treatment	P-value
**PDAS**	23.68 (13.71)	19.81 (13.99)	<0.01
**PSEQ**	32.00 (17.59)	36.60 (15.03)	<0.01
**HADS Anxiety**	6.36 (4.32)	4.78 (3.66)	<0.01
**HADS Depression**	6.74 (4.88)	5.54 (3.95)	0.02
**NRS**	5.56 (2.26)	4.26 (2.35)	<0.01

Data presented as mean (standard deviation), PDAS; Pain Disability Assessment Scale, PSEQ; Pain Self-efficacy Questionnaire, HADS; Hospital Anxiety and Depression Scale, NRS; Numeric Rating Scale,

### Univariate regression analyses

Changes in both the PSEQ (β = −0.31; 95% CI: −0.54–−0.08) and NRS (β = 0.24; 95% confidence interval [CI]: 0.01–0.47) were significantly associated with change in PDAS. Changes in HADS depression and HADS anxiety scores were not significantly associated with PDAS (Table 3).

**Table 3:** Univariate regression analysis examining predictors of change in disability.

	β (95% CI)	R^2^	F_score_	p value
**Dependent = ΔPDAS**				
**Sex**	0.11 (-0.13–0.35)	0.01	0.9	0.35
**Age**	-0.02 (-0.26–0.22)	<0.001	0.02	0.89
**Duration**	0.03 (-0.20–0.27)	0.001	0.1	0.78
**ΔPSEQ**	-0.31 (-0.54–0.08)**	0.10	7.5	0.008
**ΔHADS Anxiety**	0.20 (-0.03–0.43)	0.04	2.9	0.09
**ΔHADS Depression**	0.18 (-0.06–0.41)	0.03	2.3	0.14
**ΔNRS**	0.24 (0.01–0.47)*	0.24	4.2	0.04

n = 72;

*p<0.05,

**p<0.01

β; standardized regression coefficient, CI; confidence interval.

### Multivariate regression analyses

We used changes in PSEQ and NRS as explanatory variables in the multivariate regression analysis as an additional step to identify the most relevant factor. PSEQ (β = 0.26; 95%CI: −0.50–0.02) was associated with reduction in PDAS (R^2^_change_ = 0.12, F_change_ = 4.5). There was no association between NRS and PDAS (Table 4).

**Table 4:** Multivariate regression analysis examining predictors of change in disability.

	β (95% CI)	R^2^_change_	F_change_	p
**Dependent = ΔPDAS**		0.12	4.5 (2,69)	0.01
**ΔPSEQ**	-0.26 (-0.50–0.02)*			
**ΔNRS**	0.15 (-0.09–0.39)			

n = 72;

*p<0.05

β; standardized regression coefficient from the each step equation, CI; confidence interval.

## Discussion

There are no detailed data regarding the reason why more than half of patients did not visit our clinic again on post-treatment phase. Therefore, we cannot tell how many patients were improved and finished treatment or not improved and stopped treatment.

There were several reasons including patient’s wish, introduction to other medical institutions based on patient’s signs and symptoms, and no need for revisit because of mild pain.

We found that changes in anxiety and depression were not associated with disability, but a change in self-efficacy was associated with a reduction in disability, independent of pain severity. These findings suggest that improving self-efficacy may be an effective strategy to reduce disability among patients with chronic pain. This was consistent with the results of a previous cross-sectional study that revealed a significant association between self-efficacy and disability among Japanese patients with chronic pain [23]. The present study included Japanese outpatients with various types of chronic pain (e.g., neuropathic pain, headache, orofacial pain and visceral pain). We found that self-efficacy was a predictor of reduced disability. This was consistent with the results from previous longitudinal studies targeting musculoskeletal pain (e.g., chronic low back and shoulder pain) [17] [18] [19]. However, a 5-year follow-up study involving patients with chronic back pain showed other factors can be predictors of future disability, including sex (being female), duration of episode, and pain frequency [33]. A previous study among people with chronic low back pain reported that being a woman, pain duration, lower exercise level, and higher pain frequency appeared to play major roles in predicting disability at the 5-year follow-up [33]. In our study, sex differences and pain duration did not contribute to change in disability at the 3-month follow-up. The inconsistencies between the two studies might be attributable to differences in the follow-up period (5 years vs. 3 months) and pain site (low back vs. whole body).

Although numerous studies have reported associations between anxiety/depression and major treatment outcomes in chronic pain, such associations were not detected in the present study. This may be explained by participants in our study having less severe anxiety and depression symptoms. The mean HADS anxiety and depression scores were below the recommended cut-off values for screening anxiety and depression [27]. Although we did not control for medication, any medications (e.g., antidepressants and anxiolytics) taken before participating in this study might have reduced participants’ anxiety and depression. Further research is needed to clarify the influence of medications, especially those that affect the central nervous system.

Previous research reported that increased pain-related self-efficacy was a protective factor that helped prevent development of chronic pain [34] and had a positive influence on treatment adherence behavior [35]. These may be potential explanatory mechanisms in this study. Some previous studies demonstrated that cognitive behavioral therapy and interdisciplinary pain rehabilitation programs improved both self-efficacy and depression in patients with chronic pain [36], and suggested that activity pacing and interviews for enhancing motivation should be incorporated into clinical practice as effective therapeutic interventions [37] [38]. We did not focus on psychotherapy specifically targeting psychological factors such as self-efficacy. Therefore, it was unclear if our therapy directly affected patients’ improvement in self-efficacy. We conducted standard therapies that targeted pain reduction and improvement of ADL, according to clinical practice guidelines for chronic pain in Japan. However, there were no specific protocols for treatment. In the present study, we only described observational results and therefore cannot comment on the effectiveness of any specific therapies. Further studies are needed to identify which therapies are effective to maintain and improve self-efficacy beliefs.

Interestingly, pain duration and pain intensity were not associated with improvement of disability in this study. Improved self-efficacy may mean that patients, even those with severe persistent pain, may possibly get better and be able to maintain their social life. Measuring self-efficacy by changes in PSEQ scores may help healthcare providers manage patients’ self-efficacy in clinical practice. Our findings suggest that physicians should focus on patients’ self-efficacy as well as pain intensity, as self-efficacy may contribute to improvement in the ADL of patients with chronic pain, which in turn may help them return to their social activities. Development of therapies targeting improvement of self-efficacy may therefore be beneficial.

There were several limitations in the present study. First, this study was conducted in a single-center. A multi-centered study is needed to avoid a bias in terms of patients’ background. Second, we did not stratify patients by different chronic pain diagnoses because the number of participants was small. Third, we excluded patients who did not visit our clinic again, which might have resulted in a selection bias. More than half of the patients in the present study had not visited our clinic again at 3 months after their first visit, which might have introduced section bias. Our data did not include patients who were dissatisfied with treatment and discontinued visits to the clinic. Patients who had visited the clinic for 3 months or more were usually satisfied with their current treatment. Those patients might have shown more improvement in self-efficacy than patients who were dissatisfied with their current treatment. Fourth, this study investigated relatively few factors over a short-term (3-month) study period. Further studies are needed that explore more related factors over a longer period. Finally, we did not control therapeutic interventions, and did not use specific interventions targeting psychological factors (e.g., self-efficacy). Patients’ self-efficacy might have improved independent of therapy.

## Conclusion

Our findings provide evidence that higher levels of self-efficacy are highly correlated with greater improvements in pain-related disability. In addition to pain relief, raising self-efficacy may be a primary target in chronic pain treatment.
